# Seasonal Variation in Selected Reproductive Characteristics of Male Common Pheasants (*Phasianus colchicus*) Throughout the Annual Cycle

**DOI:** 10.3390/ani16091315

**Published:** 2026-04-25

**Authors:** Piotr Nawłatyna, Piotr Czyżowski, Sławomir Beeger, Marian Flis

**Affiliations:** Department of Animal Ethology and Wildlife Management, University of Life Sciences in Lublin, Akademicka 13, 20-950 Lublin, Poland; piotr.nawlatyna@up.edu.pl (P.N.); slawomir.beeger@up.edu.pl (S.B.); marian.flis@up.edu.pl (M.F.)

**Keywords:** common pheasant, *Phasianus colchicus*, somatic condition, reproductive activity, reproductive traits, mating vocalisation

## Abstract

Breeding in bird species inhabiting the temperate climate zone is strongly seasonally regulated. One such species is the common pheasant (*Phasianus colchicus*), widely maintained in aviary systems and of considerable importance in game management. The aim of this study was to determine seasonal variability in selected morphological traits of male common pheasants and to assess their relationship with vocalisation parameters during the annual cycle. The study was conducted on males kept under aviary conditions. Traits related to reproductive condition were analysed, including testicular mass and facial wattle height, together with mating vocalisations. The results revealed clear seasonal variation in the analysed traits. A gradual increase in body mass and spur length was observed from summer to winter, reflecting the maturation of young males and accumulation of energy reserves before the breeding season. The highest values of reproductive parameters, particularly testicular mass and wattle height, were recorded in spring, indicating peak hormonal activity and full sexual maturity. In June, a marked regression of these traits and a decline in the somatic condition traits were observed, corresponding to the end of the breeding season. Changes in vocal activity followed a similar pattern, suggesting a link between acoustic parameters and seasonal reproductive dynamics.

## Simple Summary

Seasonal changes strongly influence the biology and behaviour of many bird species living in temperate climates. One of them is the common pheasant (*Phasianus colchicus*), a species widely bred in aviary systems that plays an important role in game management. In this study, we examined seasonal changes throughout the year in several body traits linked to male fitness and reproductive activity of this species. We also recorded roosters’ mating calls to examine whether vocalisation reflects these seasonal changes. The results showed clear seasonal patterns. The highest values of reproductive traits were recorded in spring, indicating peak breeding readiness. In early summer, these traits declined, marking the end of the breeding season. Changes in mating vocalisation followed the same pattern. These findings suggest that analysis of morphometric traits, together with vocal activity, can help evaluate the condition and reproductive potential of male pheasants.

## 1. Introduction

In bird species inhabiting the temperate climate zone, reproduction shows a pronounced seasonal pattern. It is regulated primarily by photoperiod, ambient temperature, and resource availability. Seasonality affects avian physiology (e.g., gonadal development), sexual traits, and reproductive behaviours such as vocalisation and territoriality. Consequently, seasonal changes in male behaviour, morphology, and physiology may have a decisive influence on individual reproductive success.

Seasonal variation in male reproductive traits is hormonally regulated, primarily through fluctuations in androgen levels (e.g., testosterone), which influence both the development of secondary sexual characteristics and the intensity of reproductive behaviours [1]. One important physiological indicator is testis size, which varies seasonally and reflects spermatogenic activity and male reproductive investment. Testicles size is considered a reliable indicator of spermatogenic activity and, consequently, male reproductive performance [2,3]. External sexual traits also change throughout the breeding cycle. For example, facial wattles increase in size and become more intensely coloured during the mating season [1,4].

The common pheasant (*Phasianus colchicus*) is a well-established alien species in Poland, introduced from Asia in the 16th century. The biggest increase in pheasant numbers occurred in the 19th century, as a result of the popularisation of aviary breeding and numerous introductions. Today, pheasants play an important role in both ecosystems and game management [5,6,7]. The primary purpose of breeding game pheasants in Poland is to produce stock for the introduction and supplementation of wild populations. An additional use is culinary, due to the high quality of the harvested meat [8].

The species is characterised by strong sexual dimorphism and distinct territorial behaviours during the breeding season. Females are cryptically coloured, whereas males are heavier, display bright plumage colouration, long tail feathers, conspicuous red facial wattles, and long tarsal spurs [9,10,11]. Both behavioural and morphological sexual traits play a crucial role in hens’ mate choice. Females pay attention to tail length, facial wattles, and overall male appearance, as these traits provide information about male condition [1,12]. Dominant males are usually in better physical condition, as reflected in their greater resistance to parasitic infestations [13].

In Poland, breeding season lasts from April until the end of June [14], although males begin intensive mating vocalisations and territorial behaviours as early as March. The species is polygynous; male harem size usually ranges from zero to five hens. However, roosters attempt to attract as many females as possible, and some authors report harems consisting of up to ten [15,16,17] or even eighteen hens [11]. Pheasants spend most of their time on the ground, where males compete for territories and females establish ground nests. A single clutch usually contains 8 to 15 eggs [11,16].

During the breeding season, males vocalise intensively and exhibit characteristic courtship behaviours to lure hens and clearly advertise occupied territories [17]. Male vocalisations convey information about the individual’s quality and the occupied territory [11,18]. Each mating song consists of two syllables separated by a pause and is repeated at intervals of several to several dozen minutes. The structure and frequency of these vocalisations change throughout the mating season, affecting both the number of attracted females and the size of defended territories [11,14]. Analysis of mating vocalisations is a non-invasive, yet highly sensitive indicator of breeding activity, providing valuable insights into population status without disturbing the animals [19].

However, comprehensive studies on the reproduction of the common pheasant, integrating seasonal changes in morphology, physiology, and behaviour across the complete annual cycle, in both wild and farmed populations, remain relatively scarce. While some publications focus on single traits (e.g., mating vocalisations or body mass), comprehensive studies that integrate morphology and behaviour across the full annual cycle are lacking.

This study aimed to determine seasonal changes in selected reproductive-related morphological traits over the course of a complete annual cycle in common pheasants (*Phasianus colchicus*) kept under aviary breeding conditions, and to relate these changes to mating vocalisations. The research hypothesis assumed that seasonal regression of reproductive and somatic parameters would be directly reflected in the characteristics of mating vocalisations, thereby enabling acoustic analysis to serve as a non-invasive tool for assessing the physiological condition of males.

## 2. Materials and Methods

### 2.1. Study Area

The study was conducted under aviary farming conditions, with natural temperature and photoperiod, at a pheasant farm located near Lubartów (central Lublin Voivodeship, Poland) in 2024 and 2025. The production cycle at this farm is closed, meaning that replacement birds for the next breeding flock originate exclusively from the farm’s own breeding stock (from eggs produced on-site). The pheasant rearing technology applied at the farm from which the research material was obtained follows standard practices [5,20]. Chicks are obtained from eggs incubated in hatchery incubators. Until they are six weeks old, they are kept indoors. After this period, they are given access to outdoor runs, and during the autumn–winter season, they remain exclusively in the outdoor enclosures. In the autumn–winter period, the diet consists primarily of grain screenings, cereal grains, root crops, and complete feed formulated for adult pheasants.

### 2.2. Analysis of Morphometric Parameters

All studied birds hatched in May 2024 and were maintained in outdoor aviaries. At the end of each month from July 2024 to July 2025, five males were examined, resulting in a total sample size of 60 individuals. July was selected because, according to the management practices of the studied farm, all adult birds are replaced by younger individuals in June. Moreover, in July, the sex of young pheasants is already clearly distinguishable. The following traits were analysed: body mass, testicle mass and length, beak length, spur length and facial wattle size. Body mass was measured using a hanging scale with an accuracy of 5 g. Testicles were weighed using a laboratory scale with an accuracy of 0.01 g and measured along their longitudinal axis. Beak length was measured from the base of the beak to the tip. Spur length was measured at the base of the tarsus, on the inner side of the leg, at the transition between the tarsal skin and the spur. Facial wattles were measured at their maximum width, following the longest axis visible in lateral profile, from the upper margin above the eye to the lower margin below the eye. Measurements of testicles, beak, spurs, and wattles were performed using an electronic calliper with an accuracy of 0.1 mm. For all bilaterally paired traits (testicle mass and length, spur length, and facial wattle size), the mean value for each individual was calculated and used for further statistical analyses. Only post-mortem material obtained from routine farm management was used in this study. No animals were sacrificed specifically for research purposes.

### 2.3. Analysis of Acoustic Recordings

Acoustic recordings were conducted in a closed breeding aviary at the same farm, during the breeding season (from April to June), between 7:00 and 8:00 a.m. on each recording day. Vocalisations were recorded using a Zoom H1 portable audio recorder. The total recording time for each sampling date was approximately 60 min. For all vocal activity, the number of vocalisations per hour and the intervals between consecutive calls (in minutes) were determined. However, only a subset of recordings (*n* = 14) met the quality criteria required for detailed acoustic analysis. Specifically, only calls with an adequate signal-to-noise ratio and clearly distinguishable acoustic structure on the spectrogram were selected, allowing reliable measurement of the analysed acoustic parameters. The selected calls were analysed for the following time-frequency parameters: duration of the first syllable (s), duration of the second syllable (s), pause duration between syllables (s), total song duration (s), intervals between consecutive vocalisations (min), peak frequency of syllables I and II (Hz), and frequency bandwidth (Hz), measured as the difference between the minimum and maximum frequency values of the signal. All recorded material was digitally processed using Cool Edit Pro 2.0 software. For each mating call, consisting of two syllables separated by a pause, temporal parameters were determined based on oscillogram and spectrogram inspection. In contrast, the peak frequency (the frequency with the highest amplitude) was identified using Short-Time Fourier Transform (STFT) analysis. The acoustic data were treated as a supplementary component of the study and analysed descriptively to identify general patterns of seasonal variation in vocal activity at the group level. Due to housing conditions (multiple specimens kept within a single aviary), recordings could not be attributed to specific individuals. Consequently, the acoustic dataset was not used for formal statistical inference and should be interpreted with caution.

To facilitate visual interpretation of overall patterns in vocal activity, a composite descriptive index was constructed based on selected acoustic parameters. Variables were standardised (z-scores) and combined to illustrate general temporal trends in group-level vocal behaviour. This index was used solely for descriptive purposes and was not subjected to statistical inference.

### 2.4. Statistical Methods

The normality of the measured traits was assessed using the Shapiro–Wilk test, and homogeneity of variances was evaluated with Levene’s test. Due to the lack of normality, the significance of differences between months was analysed using the nonparametric Kruskal–Wallis ANOVA (H). When significant differences were detected, Dunn’s post hoc test (with Holm–Bonferroni correction for multiple comparisons) was applied to identify specific differences between pairs of months. Statistical significance was set at *p* < 0.05. To reduce the number of variables and identify the main indicators describing the biology of the studied roosters, factor analysis was applied. Factor extraction was based on the Kaiser criterion (eigenvalues > 1). Maximisation of the variance of factor loadings was performed using Varimax rotation. The results of the factor analysis were presented graphically on a factor loading plot in the (0,0) coordinate system and on box-and-whisker plots. The box-and-whisker plots illustrate seasonal variation, where the median and interquartile range (25–75%) depict the dynamics of body condition and reproductive activity throughout the annual cycle of the breeding flock (from July to June). To assess the strength of the seasonal effect on the analysed parameters, the eta-squared coefficient (η^2^_H_) was calculated for nonparametric tests using the formula: η^2^_H_ = (H − k + 1)/(N − k) [21]. Statistical analyses were performed using Statistica 13 software (TIBCO Software Inc., Palo Alto, CA, USA).

## 3. Results

Seasonal variation in the analysed somatic and reproductive parameters of male common pheasants (*Phasianus colchicus*) (Table 1) reflects both the maturation process of the breeding flock and the overlapping seasonal physiological cycle associated with reproduction. Considering July as the beginning of the production cycle, statistically significant changes were observed in the young birds until they reached full maturity in June of the following year.

The cycle begins in July with the lowest median body mass (0.92 kg) and spur length (5.1 mm), typical for juveniles from the current hatch. In subsequent months, intensive growth was observed. Body mass reached its maximum during winter (January-February: 1.5–1.6 kg), showing a significant increase compared to the summer minimum (*p* < 0.05). This likely represents an energetic reserve necessary for winter survival and preparation for the upcoming breeding season. However, from March to June, body mass did not differ significantly either from the July values or from the winter peak (Table 1). Spur length also increased steadily during this period.

In spring, the rapid development of reproductive organs and changes in external morphology were recorded. From March onward, intensive gonadal growth was observed, peaking in April and May (testicular mass: 6.89–6.57 g). Testicular size (mass and length) was significantly higher than in July starting from February/March; however, no significant decrease was observed until the end of the study period (Table 1). This was accompanied by the highest wattle height recorded during the annual cycle (May: 41.5 mm) (*p* = 0.0001); however, values recorded between October and June did not differ significantly (Table 1).

The cycle ends in June, when the flock consists of the oldest individuals (approximately 12 months old). In this month, morphometric traits such as body mass and spur length remain at relatively high levels. However, they do not differ significantly from their previously recorded maximum values (Table 1). Simultaneously, a well-marked decline in testicular mass is observed (2.90 g), signalling the termination of the breeding season. This decrease likely reflects seasonal shifts in the hormonal profile occurring after the reproductive peak.

Performed factor analysis with Varimax rotation identified two principal components explaining a total of 79.6% of the overall variability in the analysed traits (Table 2). Factor 1, termed the Somatic Condition Index, accounted for 40.0% of the variance and was strongly associated with body mass (0.92), beak length (0.78), and spur length (0.78). This component reflects overall body size and physical development. Factor 2, termed the Reproductive Activity Index, explained 39.6% of the variance and was characterised by very high loadings for testicular mass (0.98) and testicular length (0.98). This factor represents a direct indicator of seasonal reproductive activity.

Wattle height exhibited cross-loadings, correlating with both somatic (Factor 1; r = 0.58) and reproductive parameters (Factor 2; r = 0.52). Due to its slightly stronger association with the somatic component and its morphometric character, this trait was assigned to Factor 1. However, strong positive correlations between wattle height and both testicular mass (rs = 0.73; *p* < 0.001) and testicular length (rs = 0.73; *p* < 0.001) indicate that this trait is a sensitive marker of gonadal hormonal activity. The coefficient of determination (R^2^ = 0.53) indicates that more than half of the variability in this trait is directly explained by testicular growth dynamics.

**Figure 1:**
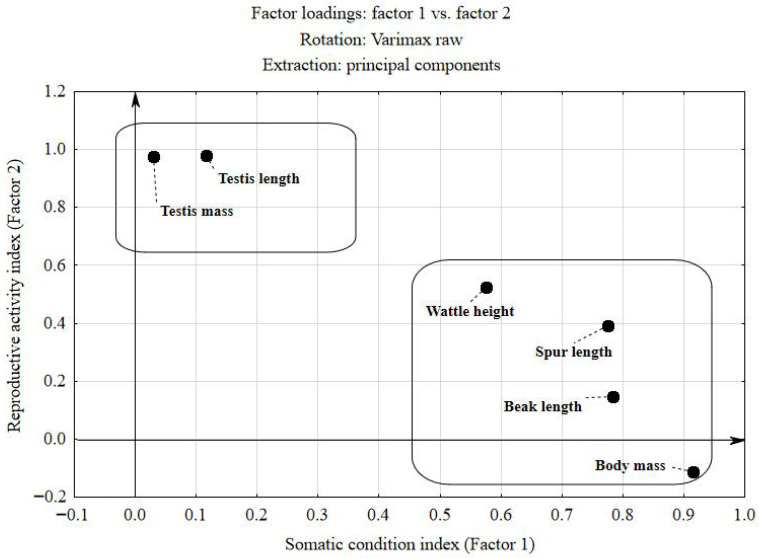
Factor loadings plot for the measured morphometric variables representing Somatic Condition and Reproductive Activity after Varimax rotation.

The factor loading plot (Figure 1) illustrates the relationship between the measured variables and the two principal components. A clear separation of traits is visible. The lower-right quadrant (Factor 1—somatic) groups traits related to body size and age (body mass, beak length, spur length). The upper-left quadrant (Factor 2—reproductive) includes testicular mass and length, demonstrating that gonadal development occurs independently of overall body mass. Wattle height occupies an intermediate position in the upper-right quadrant, supporting its dual association with both somatic and reproductive components.

The loadings plot (Figure 1) shows the relationship between the measured traits and the two main factors. The use of a coordinate system centred at 0,0 clearly reveals a division of variables into two distinct groups.

The lower right quadrant (Factor 1—Somatic condition index) includes traits related to birds’ body size and age. Body mass, beak length, and spur length are positioned close to the *X*-axis, confirming that this factor represents somatic development.

The upper left quadrant (Factor 2—Reproductive activity index) groups testicular parameters (mass and length), which are located close to the *Y*-axis. This indicates that gonadal development in pheasants is largely independent of overall body mass.

The upper right quadrant (Mixed traits) contains wattle height, which occupies an intermediate position between the two axes. This suggests that its size likely depends both on general body condition (Factor 1) and reproductive activity (Factor 2). As mentioned earlier, due to its slightly stronger association with the somatic component and its morphometric nature, this variable was assigned to Factor 1.

### 3.1. Seasonal Variation in the Somatic Condition Index

**Figure 2:**
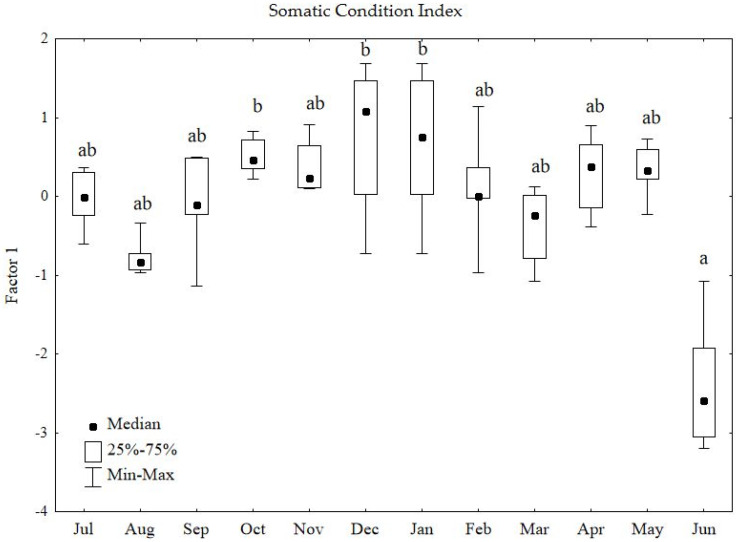
Seasonal variation in the Somatic Condition Index (Factor 1) in male common pheasants during the annual cycle. The central point represents the median, the box represents the 25th–75th percentiles, and the whiskers represent the minimum and maximum values. Kruskal–Wallis test: H = 31.90, *p* = 0.0008. Different letters (a,b) above the boxes indicate statistically significant differences at *p* < 0.05 according to Dunn’s post hoc test.

Analysis of seasonal variation in the Somatic Condition Index (Factor 1) revealed statistically significant differences in its annual pattern (Kruskal–Wallis test: H = 31.90; *p* = 0.0008) (Figure 2). The effect size was large (η^2^_H_ = 0.39), indicating that the annual cycle accounted for approximately 39% of the total variability in the physical condition of the studied males. Assuming July as the beginning of the breeding cycle, a gradual increase in somatic condition was observed in young birds, peaking during the winter period (December–January). At that time, the median index values reached their maximum levels (Figure 2), reflecting the phase of full somatic development and the accumulation of energy reserves before the breeding season.

The most distinctive feature of the cycle was a sharp decline in somatic parameters among adult males in June. This month, the median dropped significantly to its annual minimum (Figure 2, group a). This pronounced reduction indicates a substantial deterioration in physical condition following the peak of the reproductive season, clearly separating June from the winter stabilisation period (*p* < 0.05). However, post hoc comparisons indicate that these differences are limited to specific months (Figure 2).

### 3.2. Seasonal Variation in the Reproductive Activity Index

**Figure 3:**
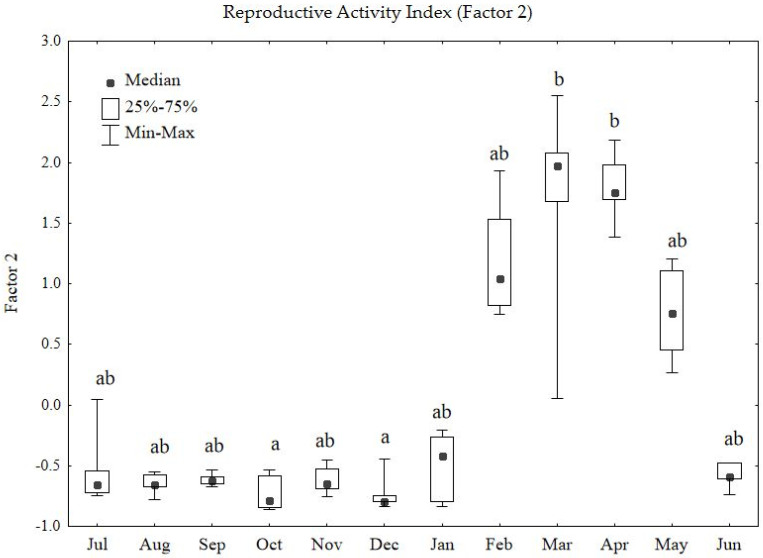
Seasonal variation in the Reproductive Activity Index (Factor 2) in male common pheasants during the annual cycle. The central point represents the median, the box represents the 25th–75th percentiles, and the whiskers represent the minimum and maximum values. Kruskal–Wallis test: H = 45.02; *p* = 0.0001. Different letters (a,b) above the boxes indicate statistically significant differences at *p* < 0.05 according to Dunn’s post hoc test.

Seasonal variation in the Reproductive Activity Index (Factor 2) showed stronger differentiation than Factor 1, as confirmed by the statistical analysis (Kruskal–Wallis test: H = 45.02; *p* < 0.0001) (Figure 3). The applied measure of effect size indicated that seasonality explained as much as 64% of the total variability of this parameter (η^2^_H_ = 0.64), demonstrating the dominant influence of the annual cycle on pheasant reproductive biology. Assuming July as the beginning of the annual cycle, the index remained at a stable, low level for most of the year (July–January). This period is characterised by sexual quiescence in young, maturing males. A rapid activation of reproductive parameters occurred in February, peaking significantly in March and April (Figure 3, group b). This high level reflects maximal testicular development and activity in the studied males, most likely associated with increased hormonal activity in preparation for the breeding season.

A key moment in the annual cycle was the abrupt regression of reproductive parameters in June, when the median fell significantly compared to the spring peak. This month, along with the autumnal period (e.g., October and December), marks the annual minimum of the index (Figure 3). This indicates that June is a period of rapid suppression of sexual activity in male pheasants. The sharp reduction in testicular size likely allows birds to reallocate resources toward the energetically demanding process of moulting and restoring reduced somatic condition.

### 3.3. Descriptive Seasonal Variation in Acoustic Parameters

A descriptive analysis of acoustic parameters suggested seasonal variation in vocal activity (Table 3). However, these observations should be interpreted with caution, as recordings were obtained from a group of birds housed together in aviary and therefore cannot be considered independent at the individual level.

A characteristic indicator of vocal activity was the interval between consecutive vocalisations, which appeared longer in June compared to April and May. No clear differences were observed in the basic structural parameters of the vocalisations during the study period, including the duration of the first syllable, pause duration, duration of the second syllable, or total call duration.

In contrast, noticeable changes were observed in frequency parameters. The peak amplitude frequency of the first syllable showed a decrease, reaching 6800 Hz in June, which represents a decline of approximately 2000 Hz compared to April (8800 Hz). A similar trend was observed for the peak amplitude frequency of the second syllable, although less pronounced. A clear narrowing of the frequency bandwidth was also observed, indicating a decline in the spectral richness of the emitted signal.

The most pronounced change was observed in the number of vocalisations. The median value declined sharply from 107.0 calls in May to only 6.0 calls in June. This abrupt decrease in acoustic activity corresponds to the previously described regression of reproductive parameters (Factor 2), suggesting that June may represent a period of territorial withdrawal and the end of the breeding season in male common pheasants.

Overall, the observed patterns indicate a seasonal decline in vocal activity and acoustic parameters, with peak values during the breeding period (April–May) and a marked reduction in June.

### 3.4. Descriptive Seasonal Pattern of Vocal Activity

**Figure 4:**
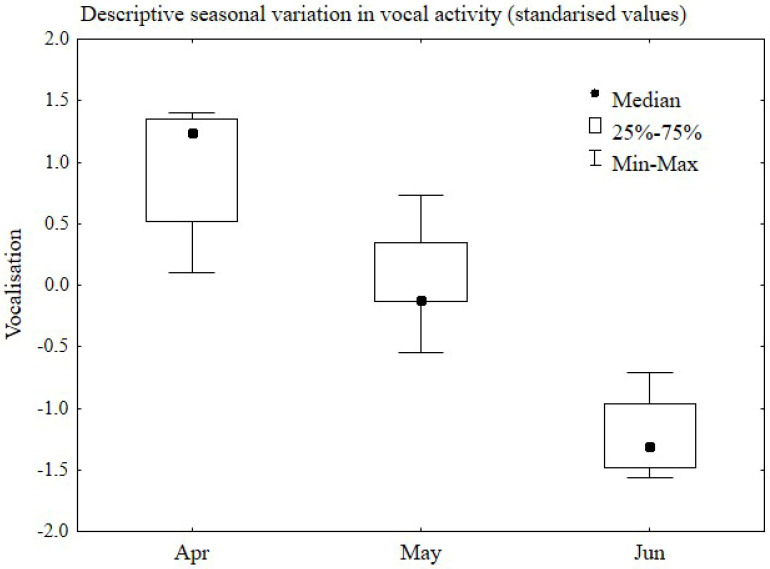
Descriptive seasonal trend of vocalisation activity in male common pheasants during the breeding season (April–June). Values are presented as standardised (z–score) medians with interquartile ranges (25–75%) and minimum–maximum values. Data should be interpreted as group-level observations.

To illustrate the overall pattern of vocal activity, a composite descriptive index based on standardised acoustic parameters was used (Figure 4). This index does not represent an independent statistical construct but serves as a visual summary of group-level trends.

A clear seasonal pattern of vocal activity was observed, as illustrated in Figure 4. The observed changes indicate a strong influence of the annual cycle on male acoustic behaviour. During the peak breeding period (April–May), vocal activity remained at a high level, reaching its maximum values, reflecting full breeding-season readiness and high male vocal performance. The median values during this period markedly exceeded the baseline levels, which is consistent with increased hormonal stimulation and high calling frequency.

A critical turning point in the annual cycle was observed in June, when vocal parameters declined rapidly. The median level of vocal activity decreased sharply, indicating a nearly complete cessation of calling behaviour. This pronounced reduction corresponds closely to the previously described regression of the testes (Figure 3). These observations suggest that June marks the period of rapid, nearly complete termination of reproductive activity in male common pheasants, as evidenced by the disappearance of mating calls.

## 4. Discussion

Studies addressing the issues discussed in the present work, particularly those concerning *Phasianus colchicus*, are relatively scarce and many are dated. Moreover, most available studies focus on a single trait measured at a single time point or over a limited period of several months, without providing a comprehensive analysis of interactions among multiple traits.

The obtained results demonstrate a gradual increase in body mass and spur length from July to the winter months, which is typical for young, growing males. This pattern indicates energy accumulation and preparation for the breeding season. It is consistent with broad patterns of seasonal body mass variation reported in birds preparing for energetically demanding life stages [22]. In contrast, the decline in the Somatic Condition Index observed in June, when the breeding season ends, reflects the high energetic costs of reproduction.

The testicular measurements confirm the influence of the breeding season on gonadal mass, as previously demonstrated [2,3,23]. Testes during the breeding season are considerably larger and heavier than in the non-breeding period. A pronounced increase in testicular weight between February and March, described by Kim and Young [2], was also observed. The rapid enlargement of testes between February and March, accompanied by a decline in body mass, indicates intensive physiological preparation for the upcoming breeding season. The maximum spring values (median > 1.8 SD) may be interpreted as representing full sexual maturity and peak hormonal activity. A sharp decline in somatic parameters in June reflects several months of intense territorial and reproductive activity and may be associated with energy-saving mechanisms. Additionally, late June marks the onset of moult, which may further deteriorate the condition. This is reflected in the pronounced decrease in the Somatic Condition Index shortly before the end of the flock’s production cycle. However, the decrease in testicular weight following the end of the breeding season reported by Kim and Young [2] occurred approximately one month later than in the present study. According to those authors, pheasants in Korea breed until late June, suggesting a longer reproductive period than observed in Poland. The present testicular measurements also correlate with elevated serum testosterone levels at the peak of the breeding season, which can therefore be expected in Polish pheasant populations as well.

The strong correlation between wattle height and reproductive parameters (Factor 2; r = 0.52) suggests that wattle height is not merely a decorative head ornament, but rather integrates information about both somatic potential and current reproductive activity, including hormonal activity and gonadal status. Wattle size was greatest from February to July, confirming its association with breeding behaviour. As reported by Papeschi et al. [4] and Baratti et al. [1], wattle size constitutes a primary cue used by females to evaluate male quality, as it is linked to circulating testosterone levels and early nutritional conditions. This enables females to select high-quality mates [1,12]. Similarly, in other galliform species, the colour and size of the wattle and comb reflect gonadal activity, testosterone levels and reproductive readiness [24,25,26,27].

Courtship vocalisation, as an indirect indicator of both somatic condition and reproductive activity, plays an important role in female mate choice [11,18]. The decline in the number of vocalisations and achieved frequencies in June closely correlates with testicular regression, indicating a relationship between hormonal status and courtship behaviour [1]. The observed patterns of vocal activity may therefore provide a preliminary indicator of reproductive activity under captive conditions. These trends may have potential applications in breeding selection, flock and wild population monitoring, and welfare evaluation. However, they require further validation under conditions allowing individual-level analysis.

It is important to note that the acoustic data presented here serve as a preliminary and descriptive supplement to our physiological findings. Due to the practical constraints of aviary breeding, where birds interact and influence each other’s behaviour, these results should be interpreted as group activity patterns. Although they provide valuable context for the seasonal peak in reproductive activity, they do not enable individual-level statistical conclusions.

The use of an aviary-based flock as the research model limits direct extrapolation of the results to wild populations. However, it enables the acquisition of a standardised dataset that would be difficult to obtain under field conditions. Studies of free-living populations in Poland are constrained by the legally defined hunting season (from 1 October to the end of February), which restricts sample collection during biologically critical periods, such as the peak of reproductive activity (May) or the rapid regression phase (June). Farm breeding ensures year-round availability of research material while simultaneously eliminating bias resulting from habitat variability or differences in diet typical of wild birds. Consequently, the data are readily accessible and highly repeatable.

A promising direction for future research would be to integrate morphometric measurements with hormonal analyses to verify the endocrine basis of the observed correlations directly. It would also be valuable to directly assess the condition of the vocal apparatus through morphometric and histological analyses of the syringeal muscles (m. syringialis). This approach would enable the determination of the extent to which seasonal variation in acoustic parameters results from physical muscle atrophy induced by declining androgen levels, and the extent to which it reflects reduced behavioural motivation to vocalise. Another promising avenue is the use of colourimetric and spectrophotometric methods to assess the saturation and intensity of facial wattle colouration. While wattle height strongly correlates with morphometric testicular development (Factor 2), the intensity of red colouration may provide independent information about the male’s current immunological condition and metabolic stress level. Such approaches could enable a more comprehensive and non-invasive assessment of welfare status in captive aviary flocks.

The results obtained may contribute to a better understanding of reproductive biology, behavioural ecology and population condition assessment in galliform birds and other avian species. They may also assist breeders in selecting appropriate traits for choosing breeding males in aviary systems and in individuals intended for later release.

### Limitations of the Study

Despite the promising results, the present study has several limitations. The analysis was based on sequential sampling of different individuals, which, despite flock standardisation, did not allow for the assessment of individual trajectories of change. A larger number of birds and continued study for a few more months would have strengthened the morphometric analyses; however, the limited sample size in each month resulted from the study being conducted on available material, which was dependent on farm management practices. This made it difficult to obtain a larger and comparable number of specimens in each month of the annual cycle. We aimed to minimise the number of individuals while ensuring sufficient statistical power to detect the expected seasonal shifts. Therefore, the relatively small sample size resulted from a compromise between material availability, study quality, and ethical aspects. Moreover, only a relatively small number of vocalisations were subjected to acoustic analysis, due to the insufficient quality of the majority of the recordings, mainly related to field recording conditions and background noise. In addition, due to the nature of aviary housing, birds were kept in a shared social environment, which led to interactions between them and prevented the attribution of vocalisations to specific individuals. Consequently, these data were treated as descriptive, group-level patterns rather than individual physiological markers. Future research under more controlled conditions, including individual-level identification, would be beneficial to validate these preliminary observations. Therefore, the relationship between morphometric traits, vocalisation, and courtship behaviour was examined only preliminarily and requires broader investigation. Finally, the lack of direct hormonal measurements limits interpretation to anatomical-behavioural associations. However, hormonal analyses are planned for the next stage of this study.

## 5. Conclusions

The present study demonstrated clear seasonal variation in morphometric traits in male common pheasant (*Phasianus colchicus*) across the annual cycle. The obtained results indicate that seasonal rhythms are the main factors regulating the biology and behaviour of the studied roosters.

At the same time, the strong association between wattle height and testicular size indicates that this trait is a reliable, visually detectable signal of reproductive readiness and reflects underlying hormonal activity.

The sharp decline in testicular mass and overall body condition observed in June marks a clear end of the breeding season, which is directly reflected in the males’ mating vocalisations. Descriptive patterns of vocal activity followed a similar seasonal trend in terms of reproductive parameters. The observed narrowing of the frequency range and the reduced calling rate in June suggest that vocal activity may reflect changes in reproductive status, although this requires confirmation under conditions allowing individual-level analysis.

While the acoustic data were treated as descriptive observations due to the social nature of aviary housing, the observed vocalisation trends complement the physiological findings, suggesting a close relationship between internal condition and behavioural expression. These observations provide a useful baseline for future research and highlight the potential value of integrating behavioural and physiological indicators into population monitoring.

These findings provide a practical and simple framework for assessing health, condition, and reproductive readiness in aviary-kept common pheasants. The integration of morphometric parameters and acoustic analysis may support breeding management, selection of males for reproduction, and monitoring of flock fitness under captive conditions.

## Figures and Tables

**Table 1:** Mean and median values of the analysed parameters [mean/median (0.25–0.75)] across the annual cycle (July–June). ANOVA Kruskal–Wallis. Different letters (a,b,c) above the values indicate statistically significant differences at *p* < 0.05 according to Dunn’s post hoc test.

Month	Body Mass [kg]	Testicular Mass [g]	Testicular Length [mm]	Beak Length [mm]	Spur Length [mm]	Wattle Height [mm]
July	0.92 ^a^ (0.8–1.0)	0.04 ^a^ (0.04–0.06)	7.5 ^a^ (7.4–7.6)	30.9 ^a^ (30.7–33.9)	5.1 ^a^ (5.1–6.0)	24.5 ^a^ (22.9–27.4)
August	1.40 ^ab^ (1.3–1.4)	0.17 ^ac^ (0.11–0.27)	11.9 ^abc^ (11.9–12.8)	37.7 ^ab^ (37.3–37.9)	7.9 ^ab^ (7.9–8.1)	31.8 ^abc^ (31.1–32.7)
September	1.27 ^ab^ (1.3–1.3)	0.11 ^a^ (0.10–0.11)	10.9 ^ac^ (9.5–11.3)	34.6 ^ab^ (34.5–34.7)	7.8 ^ab^ (6.5–8.3)	30.9 ^ac^ (29.8–33.1)
October	1.34 ^ab^ (1.3–1.3)	0.12 ^ac^ (0.12–0.12)	10.5 ^ac^ (10.1–10.6)	36.6 ^ab^ (35.5–37.7)	9.2 ^ab^ (7.5–9.4)	35.6 ^abc^ (34.6–35.6)
November	1.49 ^b^ (1.4–1.5)	0.08 ^a^ (0.08–0.08)	8.7 ^a^ (8.4–10.8)	37.5 ^ab^ (36.7–37.7)	11.1 ^ab^ (10.9–11.8)	34.0 ^abc^ (33.7–34.6)
December	1.34 ^ab^ (1.3–1.4)	0.13 ^ac^ (0.12–0.14)	9.9 ^a^ (9.5–11.1)	38.2 ^ab^ (38.0–39.7)	11.8 ^ab^ (9.5–12.2)	33.7 ^abc^ (31.8–33.7)
January	1.56 ^b^ (1.4–1.6)	0.13 ^ac^ (0.13–0.14)	11.4 ^ac^ (10.8–11.8)	38.4 ^b^ (36.9–39.6)	11.1 ^b^ (10.4–11.8)	33.9 ^abc^ (32.5–35.2)
February	1.54 ^b^ (1.5–1.6)	0.62 ^ac^ (0.58–0.98)	14.0 ^bc^ (13.5–18.0)	36.0 ^ab^ (35.0–38.0)	11.5 ^ab^ (8.1–11.8)	39.0 ^b^ (37.0–40.0)
March	1.36 ^ab^ (1.3–1.4)	6.10 ^b^ (4.55–6.21)	32.2 ^b^ (29.0–33.8)	38.0 ^ab^ (38.0–39.0)	11.8 ^b^ (11.6–12.9)	39.6 ^abc^ (35.8–40.9)
April	1.24 ^ab^ (1.2–1.3)	6.89 ^b^ (5.56–8.23)	35.5 ^b^ (32.8–36.0)	37.0 ^ab^ (36.5–37.6)	11.8 ^b^ (11.6–12.2)	40.2 ^b^ (37.7–40.6)
May	1.33 ^ab^ (1.3–1.4)	6.57 ^b^ (5.78–7.13)	35.3 ^b^ (32.5–38.0)	39.0 ^ab^ (37.4–39.9)	11.4 ^b^ (11.1–11.8)	41.5 ^b^ (40.3–42.4)
June	1.31 ^ab^ (1.3–1.3)	2.90 ^b^ (2.79–3.94)	26.1 ^b^ (21.7–30.2)	38.1 ^ab^ (38.1–38.1)	11.2 ^ab^ (11.0–11.4)	41.3 ^b^ (40.7–41.4)
*p*-value	0.0013	0.0001	0.0001	0.0020	0.0001	0.0001

**Table 2:** Factor loading matrix after Varimax rotation for morphometric traits (*n* = 65).

Variable	Factor 1: Somatic Condition Index	Factor 2: Reproductive Activity Index
Body mass	**0.92**	−0.11
Testicles mass	0.03	**0.98**
Testicles length	0.12	**0.98**
Beak length	**0.78**	0.15
Spur length	**0.78**	0.39
Wattle height	**0.58**	0.52
**Variance Explained**	**2.40**	**2.38**
**% of Variance**	**40.0%**	**39.6%**

**Table 3:** Descriptive statistics of vocalisation parameters in male common pheasants (April–June). Values are presented as mean/median (0.25–0.75).

Vocalisation Parameters	April	May	June
Intervals between consecutive vocalisations [min]	0.23/0.17 (0.14–0.35)	0.17/0.16 (0.14–0.20)	0.53/0.48 (0.36–0.70)
Duration of the I syllable [s]	0.20/0.20 (0.18–0.21)	0.20/0.20 (0.19–0.21)	0.21/0.20 (0.17–0.25)
Pause duration between syllables [s]	0.21/0.16 (0.13–0.30)	0.25/0.24 (0.20–0.30)	0.21/0.22 (0.16–0.25)
Duration of the second syllable [s]	0.18/0.16 (0.16–0.19)	0.17/0.17 (0.15–0.19)	0.14/0.15 (0.13–0.16)
Peak frequency sound amplitude of the I syllable [Hz]	8940/8800 (8400–9900)	7140/6900 (6400–7800)	7000/6800 (6300–7700)
Peak frequency sound amplitude of the II syllable [Hz]	8900/9000 (9000–10,000)	6940/6900 (6800–7200)	6900/6800 (6200–7600)
Total song duration [s]	0.59/0.59 (0.51–0.68)	0.62/0.59 (0.55–0.70)	0.56/0.55 (0.50–0.62)
Frequency bandwidth [Hz]	5590/5400 (5250–6000)	4920/4900 (4800–5000)	4163/4150 (3900–4425)
Total number of vocalisations [n]	68.0/68.0 (68–68)	107.0/107.0 (107–107)	6.0/6.0 (6–6)

## Data Availability

The datasets generated and analysed during the current study are available from the corresponding author upon reasonable request.

## References

[1] Baratti M., Ammannati M., Magnelli C., Massolo A., Dessì-Fulgheri F. (2010). Are large wattles related to particular MHC genotypes in the male pheasant?. Genetica.

[2] Kim I.S., Yang H.H. (2001). Seasonal changes of testicular weight, sperm production, serum testosterone, and in vitro testosterone release in Korean ring-necked pheasants (*Phasianus colchicus karpowi*). J. Vet. Med. Sci..

[3] Tae H.J., Jang B.G., Ahn D.C., Choi E.Y., Kang H.S., Kim N.S., Lee H.J., Park S.Y., Yang H.H., Kim I.S. (2005). Morphometric studies on the testis of Korean ring-necked pheasant (*Phasianus colchicus karpowi*) during the breeding and non-breeding seasons. Vet. Res. Commun..

[4] Papeschi A., Carroll J.P., Dessì-Fulgheri F. (2003). Wattle size is correlated with male territorial rank in juvenile ring-necked pheasants. Condor.

[5] Beeger S., Wójcik M., Flis M., Marecki M., Pyrkosz R., Dziedzic R. (2017). Cechy anatomomorfologiczne bażantów z hodowli fermowej i wolno żyjących. Med. Weter..

[6] Lavadinović V., Beuković D., Popović Z. (2019). Common pheasant (*Phasianus colchicus* L. 1758) management in Serbia. Savrem. Poljopr..

[7] Chiatante G., Meriggi A. (2022). Habitat selection and density of common pheasant (*Phasianus colchicus*) in Northern Italy: Effects of land use cover and landscape configuration. Eur. J. Wildl. Res..

[8] Kırıkçı K., Çetin O., Günlü A., Garip M. (2004). Effect of hen weight on egg production and some egg quality characteristics in pheasants (*Phasianus colchicus*). Asian-Australas. J. Anim. Sci..

[9] Górecki M.T., Nowaczewski S., Kontecka H. (2012). Body weight and some biometrical traits of ring-necked pheasants (*Phasianus colchicus*) at different ages. Folia Biol..

[10] Kayvanfar N., Aliabadian M., Ghasempouri S.M. (2015). Morphometric and morphological differentiation of the subspecies of *Phasianus colchicus* (Linnaeus, 1758) on the Iranian Plateau (Aves: Galliformes). Zool. Middle East.

[11] Iftikhar A., Yagoob I. (2024). Ecology, Behavior and Conservation Status of Ring-Necked Pheasant (*Phasianus colchicus*): A Comprehensive Review. Arch. Anim. Poult. Sci..

[12] Papeschi A., Briganti F., Dessì-Fulgheri F. (2000). Winter androgen levels and wattle size in male common pheasants. Condor.

[13] Hillgarth N. (1990). Pheasant spurs out of fashion. Nature.

[14] Czyżowski P., Nawłatyna P., Beeger S. (2024). Influence of selected parameters of mating vocalization of pheasants (*Phasianus colchicus*) on reproductive success. Pol. J. Nat. Sci..

[15] Grahn M. (1993). Mortality in the pheasant *Phasianus colchicus* during the breeding season. Behav. Ecol. Sociobiol..

[16] Göransson G., von Schantz T., Fröberg I., Helgee A., Wittzell H. (1990). Male characteristics, viability and harem size in the pheasant, *Phasianus colchicus*. Anim. Behav..

[17] Czyżowski P., Karpiński M., Beeger S., Zieliński D. (2020). Analysis of the territorial vocalization ritual of the common pheasant *Phasianus colchicus*. Acta Zool. Acad. Sci. Hung..

[18] Czyżowski P., Beeger S., Wójcik M., Jarmoszczuk D., Karpiński M., Flis M. (2022). Analysis of the Territorial Vocalization of the Pheasants *Phasianus colchicus*. Animals.

[19] Teixeira D., Maron M., van Rensburg B.J. (2019). Bioacoustic monitoring of animal vocal behavior for conservation. Conserv. Sci. Pract..

[20] Mróz E. (2012). Hodowla bażantów. Hodowla i Użytkowanie Drobiu.

[21] Fiel Peres F. (2026). Effect sizes for nonparametric tests. Biochem. Med..

[22] Kuźniacka J., Adamski M. (2010). Growth rate of body weight and measurements in pheasants reared up to the 24th week of life. Arch. Anim. Breed..

[23] Górska M., Wojciechowska J., Wojtysiak D. (2015). Sezonowe zmiany w aktywności steroidogennej oraz mikrostrukturze jąder bażanta. Rocz. Nauk. Zoot..

[24] Mougeot F. (2008). Ornamental comb colour predicts T-cell-mediated immunity in male red grouse Lagopus lagopus scoticus. Naturwissenschaften.

[25] Lengyel K., Rudra M., Berghof T.V., Leitão A., Frankl-Vilches C., Dittrich F., Duda D., Klinger R., Schleibinger S., Sid H. (2024). Unveiling the critical role of androgen receptor signaling in avian sexual development. Nat. Commun..

[26] Qi K., Amevor F.K., Liu Z., He J., Xu D., Zhai C., Wang Y., Wu L., Wang Y., Shu G. (2025). Relationship between comb development, immune regulation, growth hormone, testosterone, and growth traits in Tianfu broilers. Poult. Sci..

[27] Zuk M., Thornhill R., Ligon J.D., Johnson K., Austad S., Ligon S.H., Wilmsen Thornhill N., Costin C. (1990). The role of male ornaments and courtship behavior in female mate choice of red jungle fowl. Am. Nat..

